# Regulation of nursing professionals in Cambodia and Vietnam: a review of the evolution and key influences

**DOI:** 10.1186/s12960-019-0388-y

**Published:** 2019-07-03

**Authors:** Noriko Fujita, Sadatoshi Matsuoka, Kyoko Koto-Shimada, Megumi Ikarashi, Indrajit Hazarika, Anthony B. Zwi

**Affiliations:** 1 National Center for Global Health and Medicine, 1-21-1 Toyama, Shinjyukuku, Tokyo, 162-8655 Japan; 2 The University of Tokyo, 7-3-1 Hongo, Bunkyoku, Tokyo, 113-0033 Japan; 3 Regional Office for the Western Pacific, World Health Organization, Manila, Philippines; 4 Health Rights and Development (HEARD@UNSW), School of Social Sciences, Faculty of Arts and Social Sciences, University of New South Wales, Sydney, NSW 2052 Australia

**Keywords:** Nursing profession, Professional development, Regulatory framework, Professional mobility, Cambodia, Vietnam, ASEAN, ASEAN Mutual Recognition Arrangement

## Abstract

**Background:**

In 2006, the countries of the Association of Southeast Asian Nations (ASEAN) signed the Mutual Recognition Arrangements (MRA) in relation to nursing services in the region. This agreement was part of a set of policies to promote the free flow of skilled labor among ASEAN members and required mutually acceptable professional regulatory frameworks.

This paper presents a narrative review of the literature to (1) describe progress in the development of the regulatory framework for nursing professionals in Cambodia and Vietnam since 2000 and (2) identify key factors, including the MRA, that affect these processes.

**Methods:**

For document review, policy documents, laws, regulations, and published peer-reviewed and gray literature were reviewed. Data were triangulated and analyzed using a tool developed by adapting McCarthy et al.’s regulatory function framework and covering eight functions (legislation, accreditation of preservice education, competency assessment, registration and licensing system, tools and data flow of registration, scope of practice, continuing professional development, professional misconduct and disciplinary powers).

**Results:**

Cambodia and Vietnam have made remarkable progress in developing their regulatory frameworks for nursing. A number of key influences contributed to the development of nursing regulations, including the signing of the MRA in 2006 and the establishment of the Joint Coordinating Committee on Nursing (AJCCN) in 2007 as key milestones. Macroeconomic and political factors affecting the process were economic growth and an emerging private sector, social demand for quality care and professionalism, global attention to health workforce competencies, the role of development partners, and regular monitoring and mutual learning through AJCCN. A period of incubation enabled countries to develop consensus among stakeholders regarding regulatory arrangements; this trend accelerated after 2010 by bringing national regulatory schemes into conformity with the regional framework. Some similarities in the process (e.g., preservice education first, legislation later) and differences in key actors (e.g., professional councils and the capacity of nursing leaders) were observed in two countries.

**Conclusion:**

Further development of the regulatory framework will require strong nursing leadership to sustain achievements and drive continued progress. The adapted tool to assess regulatory capacity works well and may be of value in assessing the development of regulations in the nursing profession.

**Electronic supplementary material:**

The online version of this article (10.1186/s12960-019-0388-y) contains supplementary material, which is available to authorized users.

## Background

Universal health coverage (UHC) is the key to achieving the health-related Sustainable Development Goals (SDGs). UHC will require an equitably distributed, qualified and motivated health workforce [1, 2] to deliver effective services across the life course. In low- and middle-income countries (LMICs) [3], the uneven distribution and low quality of health workers remains a persistent constraint, attributable to a range of factors such as limited training opportunities, poor working and living conditions in rural areas and concentration of income-generating opportunities in urban settings (e.g., through secondary employment) [1]. In addition, globalization and the resultant liberalization of labor markets have accelerated transnational movements of health workers [4]. Nurses constitute a large proportion of these migratory flows [3, 5].

In 2006, the members of the Association of Southeast Asian Nations (ASEAN) signed the Mutual Recognition Arrangements (MRA) for a range of key professions, including nursing. This agreement is part of a desire to facilitate the free flow of goods, services, investment, capitals, and skilled labor in ASEAN. The objectives of the MRA were to facilitate the mobility of nursing professionals, exchange information and expertise on standards and qualifications, promote the adoption of best practices in professional nursing services, and provide opportunities for the capacity building and training of nurses within ASEAN [6].

According to the MRA, a nurse refers to a person who has completed the required professional training and has been assessed by the Nursing Regulatory Authority in the source country. In addition, nurses should be ethically and legally qualified to undertake professional nursing practice and be registered and/or licensed by the regulatory authority of the country of origin. For nurses to practice in the recipient country, they have to be registered or licensed with the relevant regulatory authority [6].

To ensure the competency of health workers and safeguard the quality and safety of health services, countries are required to establish an appropriate regulatory framework [6]. The role of regulatory authorities has become increasingly important given heightened migration, part of an effort to ensure that qualified health workers from source countries can meet the regulatory requirements and find gainful employment in the receiving country [6]. In ASEAN countries, efforts are underway to strengthen the nursing regulatory environment for health workers, with the objective of improving the quality and safety of health services. This ambition has intensified over the past decade, and in Cambodia and Vietnam (the focus of this paper), actions have been initiated to strengthen the regulatory frameworks for nursing professionals. However, there is limited documentation of the context in which this effort has been occurring, the influences upon it, and the processes through which the regulatory framework has unfolded.

This paper presents a narrative review of the literature to (1) describe progress in the development of the regulatory framework for nursing professionals in Cambodia and Vietnam since 2000 and (2) identify the key factors that have affected the process, including the MRA.

## Methods

### Concept of regulatory framework

**Fig. 1:**
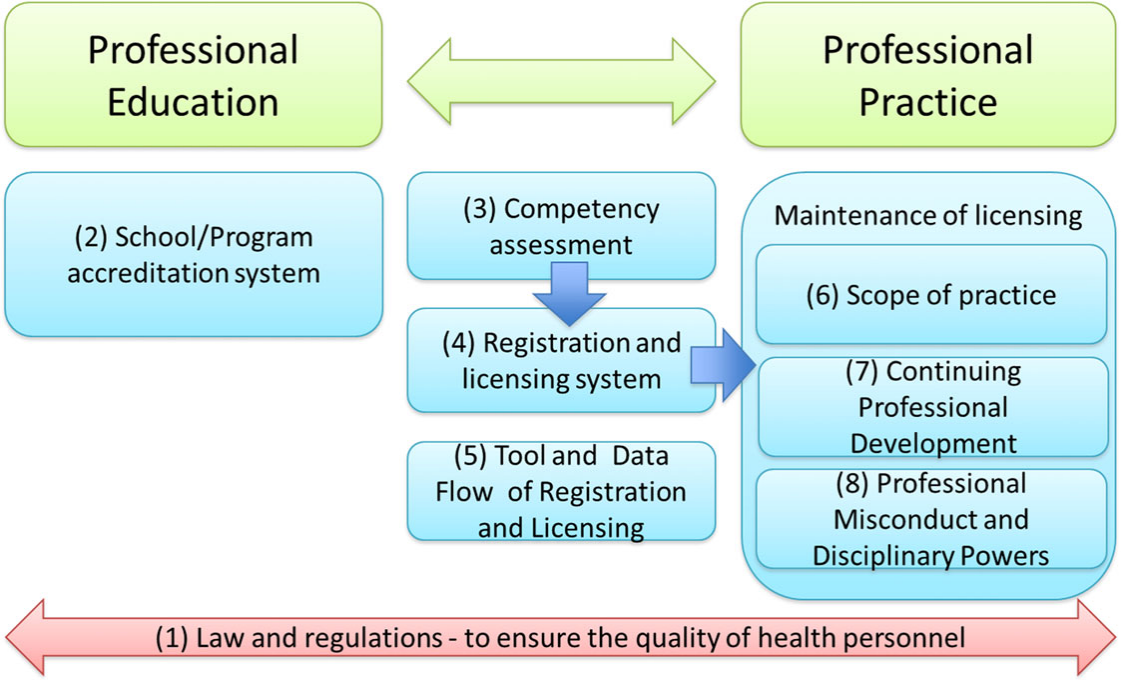
Concept of regulatory framework. Authors’ adaptation of the Regulatory Board Governance Toolkit

The concept of the regulatory framework used in this paper is adapted from the Regulatory Board Governance Toolkit [7] and covers diverse regulatory functions, ranging from the enhancement of preservice education through continuous professional development (Fig. 1). The adaptation takes into account existing in-country practices. For instance, school or program accreditation mechanisms, including national examinations, have been introduced to ensure that nurse graduates have acquired the required competencies to practice. In addition, registration and licensing systems are direct measures used to control entry into the profession. The knowledge and skills of nurses are to be regularly updated through continuing professional development (CPD) opportunities, which also form the basis for the periodic renewal of licenses. To further improve the quality of health services, codes of ethics, standards, and scopes of practice (SOP) have been instituted. The earlier toolkit of the Regulatory Board Governance Evolution Toolkit was used for our adaptation, as it is being modified to help capture elements of these evolutionary processes. [7]

### Data collection

The authors reviewed policy documents, laws, and regulations in Vietnam and Cambodia in relation to nursing education and professional nursing practice. To capture all regulatory functions of nursing professionals, the relevant peer-reviewed and gray literature published between 2000 and 2017 in three languages (English, Khmer, and Vietnamese) were also reviewed by authors. Prior to analyzing content, all documents were translated into English. Databases (PubMed and Google Scholar) were searched using key search terms such as nursing professional, regulatory framework, and ASEAN to identify all relevant papers.

### Data analysis

Collected information was synthesized to produce a chronologic overview of advances in regulatory functions during the period 2000–2017 (Additional files 1 and 2). An adapted version of the regulatory function framework (RFF) (Table 1) developed by McCarthy et al. [8–10] and the Regulatory Board Governance Toolkit [7] was used to objectively assess advances in core regulatory functions [9]. The assessment was validated through published and synthesized data and triangulated with insights from those authors based on their field experience working on nursing administration and education in Cambodia and Vietnam.

**Table 1:** Regulatory function framework

No.	Function	Stage 0	Stage 1	Stage 2	Stage 3	Stage 4
1	Nursing legislation	Key issues of legislation are not identified.	Consensus among stakeholders around whether a new legislation or amendments to existing legislation are needed. Legislation drafted with stakeholders.	Legislation approved and publication of the legislation. Dissemination of the legislation to and provision of trainings for implementers.	Implementation of the legislation.	Monitoring and evaluation of compliance and impact. Review and revision of the legislation according to regional or global standards.
2	Accreditation of pre-service education	School accreditation system is not available.	Standards for accreditation of nursing schools are developed.	Nursing schools/programs are accredited by a regulatory authority through initial assessment.	Assessments are regularly carried out by a regulatory authority. Various levels of accreditation granted (i.e. probationary, conditional)	Accreditation standards align with regional or global guidelines. Accreditation status available to the public.
3	Competency assessment	National competency standards are not available.	National competency standards are being developed.	Examination or assessment content meets national competency standards.	An examination or assessment process is in place for initial registration and licensure.	Examination content aligns with global guidelines or regional competency standards and is updated regularly.
4	Registration and licensing system	Registration and licensing is not legally required for nurses to practice.	Registration and licensing is legally required for nurses to practice. Renewal of registration or licensing is required at intervals specified by the regulatory authority.	Registration and licensing system includes public sector nurses working under the Ministry of Health.	Registration and licensing system includes all public and private sector nurses.	Registration database can exchange with other health information system. Registration data used by decision makers for workforce policy and planning.
5	Tool and data flow of registration and licensing	Registration is not required for nurses to practice. But data on nurses working in the public sector are available (e.g. number of nurses).	The register is primarily paper-based system. Data are collected through a provincial level.	Both paper and electronic system for registration is used. Data are collected and gathered at national level.	Registration system is completely electronic and available online.	Registration, licensure and re-licensure services are available online. Data displays various registration statuses of nurses.
6	Scope of practice (SOP)	SOP not defined by regulation.	SOP are under development by a regulatory authority.	Nationally standardised SOP for all nurses categories are developed.	Nationally standardised SOP for all nurses categories are implemented.	The SOP are regularly and systematically reviewed and revised. All SOP align with regional or global guidelines and standards for nursing.
7	Continuing professional development (CPD)	CPD is voluntary or is provided on ad-hoc basis.	CPD is officially required for renewal of registration or licensure.	National CPD framework for nursing is developed.	National CPD framework for nursing is implemented.	System in place to monitor CPD compliance. Regular evaluation of CPD program carried out. CPD content aligns with reginal or global guidelines. Penalties for non-compliance with CPD exist.
8	Professional misconduct and disciplinary powers	Standards of professional conduct are not defined.	Standards of professional conduct are defined by a regulatory authority.	A regulatory authority investigates or initiates inquiries into professional misconduct. Appeals processes are available and accessible.	The processes and documentation of complaints and sanctions is transparent. Processes are in place for member of the public to lodge a complaint.	Professional conduct standards align with regional or global guidelines. The complaint management process is regularly evaluated for transparency and timeliness. Information on complaints and sanctions is available to the public.

The adapted RFF comprised eight regulatory functions and five progressive stages. The eight functions are (1) nursing legislation, (2) accreditation of preservice education, (3) competency assessment, (4) registration and licensing system, (5) tool and data flow of registration and licensing, (6) SOP, (7) CPD, and (8) professional misconduct and disciplinary powers. The stages range from nonexistent or minimal (Stage 0) to high (Stage 4) capability and are defined in line with regional or global recommendations (Table 1). Each stage is characterized by key features that contribute elements and at times prerequisites for advancing to the next stage [10]. The authors analyzed the chronological progress of regulatory development and determined the stage that best characterized the status of each regulatory function in 2000 (start year of analysis) and in 2017. Data were contextualized to identify factors that contributed to the process, progress and evolution of the regulatory framework, including but not limited to the ASEAN MRA.

## Results

**Fig. 2:**
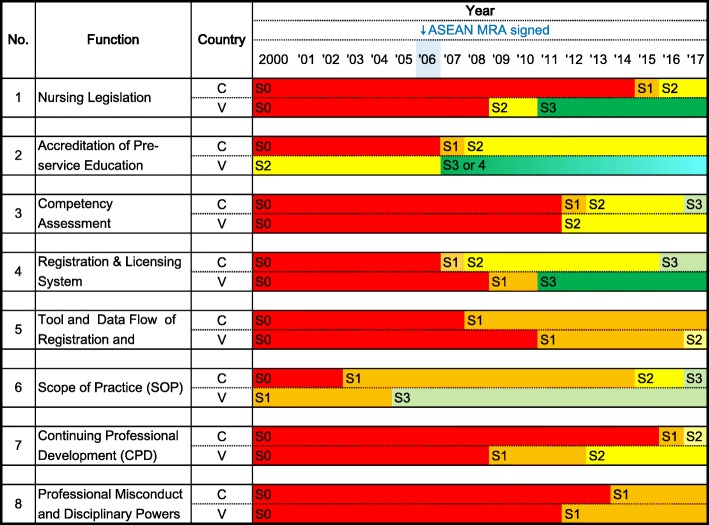
Comparison of progress in the regulatory framework development for nursing professionals in Cambodia and Vietnam

Figure 2 shows progress in regulatory framework development for nursing professionals (2000–2017) in Cambodia and Vietnam. Both countries made remarkable progress that has accelerated in recent years.

### Progress in the regulatory framework development

#### Cambodia

As a result of the country’s civil war and genocide, the 1990s revealed a critical shortage of health workers in administration, education, and clinical settings in Cambodia [11]. Since then, political stability and an enabling policy environment have stimulated economic growth. In 1999, the country joined ASEAN.

Given its youthful population, health and education had become key development priorities [12]. Education was also identified as offering opportunities for investment; the number of private nursing institutions increased from one in 2003 to 12 by 2016. Only six of these institutions were under the control of the Cambodian Ministry of Health (CMOH) [13]. In 2000, the CMOH was the main stakeholder for regulating the quality of health professionals and services, and all eight functions were minimally developed (Stage 0). The accreditation of preservice education (Function 2) and competency assessment (Function 3) were significant concerns.

Growing concerns regarding the competency of health workers resulted in the adoption of the Royal Decree on Accreditation of Higher Education in 2003. This decree was followed by Sub-Decree 21 on Health Education in 2007, which stipulated conditions and criteria for the accreditation of educational institutions for health professionals in public and private sectors and the establishment of national entrance and exit examinations for higher education. The standard nursing curriculum was developed as a 3-year Associate Degree of Nursing (ADN) and 4-year Bachelor of Science in Nursing (BSN) in 2007 and 2008, respectively. The curricula outlined minimum standards, including admission requirements, study duration, and educational content (Stage 1). Based on these standards, since 2008, the CMOH has authorized the educational programs of each school (Stage 2). The Accreditation Committee of Cambodia, the authority accrediting all higher education institutions, comprises relevant departments of the CMOH and Ministry of Education, Youth and Sport. It issued nine minimum quality standards for the accreditation of higher education. These standards, however, do not cover important aspects of clinical education and training, such as teacher to student ratio [14]. In 2017, regular audits of the schools and educational programs were still not undertaken on a consistent basis.

Regarding Function 3, the Core Competency Framework for New BSN Graduates was developed in 2012 to guide preparations for the National Exit Examination (Stage 1), which started in 2013 and governed the quality of education in the public and private sectors (Stage 2). By 2016, the CMOH started National Entrance and Exit Exams for all bachelors and associate degrees in all 16 health professional disciplines. The examination was not yet used as the basis for registration and licensure in 2017 (Stage 3).

In 2007, the Cambodian Council of Nurses (CCN) was established by Royal Decree. This Decree required that nurses be registered to practice (Stage 1 in Function 4) and was implemented in the public sector in 2008 (Stage 2 in Function 4). Despite the commencement of registration, the number of registered nurses did not increase as expected. The CCN could not take on all expected regulatory functions due to limited capacity. The CMOH is responsible for the accreditation of preservice education and competency assessment. Both CMOH and CCN utilized the limited availability of technical support from development partners to advance their capabilities. In 2003, CMOH issued the SOP for hospital nurses (Stage 1 in Function 6), defining task-based nursing activities following the French approach, but this SOP was not fully introduced in clinical settings. It was updated in 2015 (Stage 2) along USA professional lines [15]. Although the updated SOP described all categories of nurses (i.e., primary nurse, ADN, and BSN), it is not fully aligned with either the curricula or the competency framework (Stage 3).

In 2016, an iatrogenic outbreak of human immunodeficiency virus (HIV) infection in a rural province created the political momentum to further strengthen the regulatory framework, resulting in the passage of the Law on Regulation for Health Practitioners [16]. This was a breakthrough in accelerating regulatory framework developments (Stage 2 in Function 1), as it led to the adoption of comprehensive legislation for all health professionals in the same year (Stage 1 in Function 1) [17, 18]. This Law on Regulation established registration and licensure and their renewal as mandatory requirements for all practicing health professionals including nurses (Stage 3, Function 4). Since the adoption of this Law on Regulation, the CCN has benefitted from a joint secretariat created to support the administrative functions of the five health professional councils.

The registration function has been strengthened, and by 2017, 68% of the nurses (predominantly working in the public sector) had been registered with CCN (Stage 3, Function 4) [19]. However, registration is somewhat inefficient and is paper-based, with data from the provinces collected and sent to the national level (Stage 1, Function 5).

In the absence of a systematic approach, CPD opportunities continue to be ad hoc (Stage 0, Function 7) [14, 19, 20]. By 2017, a framework for CPD was being developed in relation to the maintenance of the professional license to practice (Stage 2). The CCN is expected to monitor professional misconduct according to the Code of Ethics for Nurses, but was not fully functional by 2017 (Stage 1, Function 8).

#### Vietnam

In 1986, a policy of “renovation” (*Doi Moi*) was introduced to overcome the economic problems of the 1980s. This policy led to dramatic social and economic changes, including in the health and education sectors [21]. The country became an ASEAN member state in 1995 [21].

In 1998, an Education Act was enacted that stipulated standards for all educational levels (primary, secondary, and tertiary) and types (general and vocational) under the responsibility of the Vietnamese Ministry of Education and Training (VMOET). The VMOET was able to accredit 2- and 3-year courses as of 2000 (Stage 2, Function 2).

With an emphasis on improvement of skilled health workers to drive economic growth, in 2005, the Law on Education was amended to strengthen the relationship between education and job categories and to encourage private funding and investment in the educational sector [22]. The number of nursing schools increased from 70 in 2005 to 150 in 2015, including a growing number of private schools [23]. Similar to Cambodia, concerns regarding the quality of training in the higher education system became evident with the rapid expansion of higher education institutions, particularly those in the private sector. Based on the Act amended in 2005, the VMOET issued a Circular defining the standard curricula for vocational education [i.e., secondary schools (2-year course) and colleges (3-year course)] in 2010 and higher nursing education [i.e., universities (four-year course)] in 2012. According to the Law on Education (amended in 2009) and the Law on Vocational Training (amended in 2014), two Ministries were involved in Function 2. University education is accredited by the VMOET, and education at secondary schools and colleges is accredited by the Vietnamese Ministry of Labor, Invalids and Social Affairs (VMOLISA). The VMOET and VMOLISA have been responsible for monitoring the implementation of standard requirements (Stage 3, Function 2). The Vietnamese Ministry of Health (VMOH) has been responsible for reviewing the content of educational curricula since 2000 and has established and monitored the CPD system.

With an intent to further improve quality and safety of health services, in 2009, the Law on Examination and Practice was drafted, stipulating the need for health professionals to be registered and licensed (Stage 2, Function 1). This law was implemented in 2011 (Stage 3, Function 1), and registration and licensing functions were consolidated in both public and private sectors (Stage 3 since 2011, Function 4). However, standard curricula and programs for clinical training are still not fully developed. The process for license renewal was also not defined in the Law on Examination and Practice. As of 2017, paper- and electronic-based registration systems were introduced, but comprehensive data were not available at the central level (Stage 2, Function 5).

In 2009, competency standards were drafted by the Vietnamese Nursing Association (VNA) and issued in 2012 by the VMOH. However, as of 2017, the competency standards were not fully reflected in educational programs of vocational and higher education and nursing practices performed at clinical sites (Stage 1, Function 3). A regulation on competency assessment was under discussion as of 2017.

Regarding Function 6, a Scope of Work and Salary Scale for All Public Nurse Categories existed in 2000 (Stage 1); it was revised and implemented in 2005 (Stage 3) and linked to the nurses’ salary scale. SOP was defined for all nurse categories based on educational curriculum, but actual SOP performed at clinical sites did not yet correspond to nurses’ categories as of 2017.

The CPD requirement is defined in the Education Act amended in 2005. In 2009, national CPD guidelines for health professionals were developed by the VMOH. CPD was provided at hospital levels [24] but not completely linked to the licensure and registration process. Following the execution of the Law on Examination and Practice, in 2013, CPD guidelines were amended to make CPD an official requirement for licensure (Stage 1, Function 7). VMOH and VNA developed the code of ethics in 2012. The conditions for the revocation of the license are mentioned in the Law on Examination and Practice (Stage 1, Function 8) but have not been fully implemented.

As of 2017, all regulatory functions were managed by the VMOH, except for Function 2, which is under the VMOET and VMOLISA.

### Key factors affecting the evolution of these regulatory processes

Based on the review of relevant information and the authors’ understanding of country context in both countries, the following were identified as key influences in advancing the evolving regulatory framework and functions.

#### Economic growth and an emerging private sector

In both countries, economic reforms and steady growth have resulted in a growing private sector, manifested by an increased number of private schools, hospitals, and clinics in the health sector [14, 25]. Continued concerns regarding the quality and safety of services contributed to the need to regulate the private sector [2, 26]. These concerns have been an important driving force for the establishment of quality assurance mechanisms, such as the accreditation of preservice training (Function 2).

#### Social demand for quality care and professionalism

Consumer awareness has enhanced accountability and increased pressure on governments and health workers [2, 27, 28]. In addition, adverse medical events following treatment have attracted negative media attention. These events may also have contributed to the development of a regulatory framework (e.g., cluster of HIV cases triggered by an unqualified health worker in Cambodia [29]). Under these circumstances, increased social demand for quality care has, in part, driven progress toward strengthening regulatory frameworks (accelerating Function 1).

#### Global attention to health workforce competencies

Global attention to developing a competent health workforce has also influenced regulations [30, 31]. Nursing leaders have been actively engaged in bringing this issue to the attention of policy-makers [32].

In Vietnam, the 5-year Health Plan 2011-2015 focused on the quality and quantity of health workers (accelerating Functions 2, 4, and 5). In addition, specific policy directions and documents for strengthening the nursing and midwifery workforce have been issued since 2002 (accelerating Functions 2, 6, 7, and 8) [33].

In Cambodia, a series of Health Workforce Development Plans have been developed and updated since 1996. Each plan was based on contextual priorities; the first plan focused on the quantity of the health workforce, the second on the quality of the health workforce (accelerating Functions 2 and 3), and the current third plan also focuses on quality (accelerating not only Functions 2 and 3 but also Functions 4, 5, 6, 7, and 8). However, unlike Vietnam, there are no specific policy directions for the nursing and midwifery workforce, even though the Plans emphasize their importance in the context of health service in the country.

#### Role of development partners

In both countries, development partners have played an important role in supporting the development of the regulatory system. Inputs by technical advisors and financial support by the WHO and bilateral agencies (from Japan, USA, Germany, Australia) have contributed to progress in the different functions. In addition, direct technical support at the institutional level (e.g., from universities and associations from Australia, Canada, and South Korea) has been an important factor in Vietnam [34–36]. However, patchy development of some components of the regulatory system was also apparent. For instance, the development of SOP (Function 6) in 2003 was undertaken without connecting it to other functions in Cambodia, the development of competency (Function 3) was undertaken without linkage to educational programs (Function 2), and developing the code of ethics (Function 8) was undertaken without clarifying how it will be implemented in both countries.

#### Regular monitoring and mutual learning through AJCCN

The ASEAN Secretariat recognized the disparity in maturity of the nursing regulatory framework between countries. To maximize benefits from integration, ASEAN recognized the need for technical and development cooperation to address gaps and accelerate economic integration with less-developed ASEAN member states [37, 38].

In 2007, the ASEAN Joint Coordinating Committee on Nursing (AJCCN) was established, a year after the ASEAN MRA was signed. The AJCCN comprised representatives from nursing regulatory authorities in each country. The AJCCN facilitates the MRA on nursing services. Regular monitoring by the AJCCN, the CCN, and relevant departments of the MOH (both Cambodia and Vietnam) has promoted mutual learning among ASEAN countries and contributed to developing a common core competency framework (Function 3) and SOP (Function 6) [39, 40].

## Discussion

This paper describes the evolution of the regulatory framework for nursing professionals in Cambodia and Vietnam. Between 2000 and 2017, both countries made substantial progress. The signing of the MRA in 2006 and the establishment of the AJCCN in 2007 were important milestones, occurring as they did within the context of changing macroeconomic and political developments in both countries. Multilateral action was followed by a period of incubation during which each country focused on building consensus among the different stakeholders involved. Further developments in regulatory functions have accelerated since 2010. Several factors have contributed to this progress and evolution, including a growing government commitment along with social demand for improved quality and safety of health services. Countries have also initiated regulatory processes to ensure conformity of national regulatory schemes with the framework set out by the ASEAN MRA. Regular monitoring by the AJCCN along with support from development partners has encouraged progress.

Cambodia and Vietnam have moved in a similar direction and have followed a similar sequence of functional development. This development has built upon preservice education (Function 2), followed by overarching legislation (Function 1) to license health professionals. In both countries, economic growth accompanied by a dramatic increase in private institutions in health and education were early triggers for establishing a system able to regulate the quality and performance of the growing number of health professionals.

Early on, the regulatory systems were somewhat premature and incoherent; over time, consensus among stakeholders, including the political establishment in each country, has been built. These changes have strengthened the regulatory system. Unlike Malaysia [41, 42], Singapore [42, 43], and Thailand [42, 44], which set out legislation for each specific cadre of health professionals, both Cambodia and Vietnam promoted broad overarching legislation covering regulation of all health professions, including nursing. Such overarching legislation stimulated a more coherent and comprehensive approach.

The structure of the regulatory agency differs between the two countries. In Cambodia, five health professional councils were established. In Vietnam, the Ministry of Health is responsible for implementing regulatory functions. These efforts have been supported by the VNA, which played an important role in developing a code of ethics and core competencies as well as in implementing CPD [25]. The revision of the Law on Examination and Treatment (2009) is still under discussion in 2018 and may form the basis for the establishment of a professional council.

Regardless of the regulatory authority, in both countries, the capacity to implement these important regulatory functions has been recognized as challenging to achieve. Limited degrees of leadership in nursing have been one of the biggest constraints [45, 46], and both countries acknowledge the need to invest in increasing the quantity and quality of nursing leaders who can advance implementation and link all the necessary regulatory functions [47] into a more coherent whole.

Although the ASEAN MRA is intended to facilitate the mobility of nursing professionals, limited data on supply, demand, and movement of professionals make it difficult to quantify actual skill mobility [48]. However, prior reports, anecdotal evidence, and the authors’ country-level experience suggest that the actual flow of professional nurses among Cambodia, Vietnam, and other ASEAN countries is still limited. This situation may be due to identified challenges, such as the diversity of education and training, licensing requirements, language, and cultural dimensions of clinical practice [1]. Once the MRA becomes fully implemented, however, it could create the opportunity for professionals from ASEAN countries to become more mobile and gain significant skills and knowledge [49]. However, the MRA also creates the risk of increased transnational flows, especially from resource-constrained countries to those that are already better resourced [4]. In pursuit of the WHO Global Code of Practice on the International Recruitment of Health Personnel, ASEAN countries will need to consider promoting voluntary principles and practices for the ethical international recruitment of health personnel [50].

This paper has set out to share insights regarding the evolution of the regulation of nursing professionals and how this has progressed in Cambodia and Vietnam over the past two decades. Our findings are presented by adapting an earlier tool to delineate how the regulatory framework functions (RFF) have evolved; we call our adaptation of the Regulatory Board Governance Evolution Toolkit to document the evolution of functions and stages in nursing regulation. The paper has some limitations that should be noted. First, the paper is based on a review of publicly available policy documents and legislation as well as insights from key informants. Second, although the RFF used in the paper is helpful in assessing progress, the validity of the results could have been further enhanced by engaging a larger number of stakeholders. The RFF is also limited in its capacity to capture nuanced advances or smaller steps within the stages and functions [51]. These would require further in-depth policy and qualitative analyses. In addition, the RFF is unable to fully demonstrate the connections among the regulatory functions. The RFF did not incorporate certain functions, such as leadership and management, which are known to be important in advancing regulatory functions.

## Conclusion

In Cambodia and Vietnam, remarkable progress has been made in developing the regulatory framework for nursing professionals. Since 2000, key legislation has been enacted in both countries, which has facilitated the introduction of the main regulatory functions. A number of these functions are still at an initial stage, while others have evolved considerably. However, the political commitment to provide universal access to quality and safe health services has provided new momentum to further strengthen these regulatory systems and frameworks. In addition, the growing social demand for improved professional services and quality of care has contributed impetus. Furthermore, this is all underpinned by the multicounty commitment to the ASEAN MRA, with the AJCNN seeking to both create an enabling environment and monitor progress. Nevertheless, there is a recognized need for a new generation of nurse leaders, a set of professional cadres underemphasized through these regulatory functions: it is their leadership that will be required to capitalize on the momentum and drive the further development of the regulatory framework.

## Additional files

Additional file 1: Chronologies of nursing regulatory development in Cambodia. (XLSX 15 kb)

Additional file 2: Chronologies of nursing regulatory development in Vietnam. (XLSX 12 kb)

## Data Availability

All data generated or analyzed during this study are included in this published article and its Additional files.

## Abbreviations

ADN: Associate Degree of Nursing
AJCCN: Joint Coordinating Committee on Nursing
ASEAN: Association of Southeast Asian Nations
BSN: Bachelor of Science in Nursing
CCN: Cambodian Council of Nurses
CMOH: Cambodian Ministry of Health
CPD: Continuing professional development
HIV: Human immunodeficiency virus
LMICs: Low- and middle-income countries
MRA: Mutual Recognition Arrangements
RFF: Regulatory function framework
SDGs: Sustainable Development Goals
SOP: Scope of practice
UHC: Universal health coverage
VMOET: Vietnamese Ministry of Education and Training
VMOLISA: Vietnamese Ministry of Labour, Invalids and Social Affairs
VMOH: Vietnamese Ministry of Health
VNA: Vietnamese Nursing Association
